# Advancing crested wheatgrass [*Agropyron cristatum* (L.) Gaertn.] breeding through genotyping-by-sequencing and genomic selection

**DOI:** 10.1371/journal.pone.0239609

**Published:** 2020-10-08

**Authors:** Kiran Baral, Bruce Coulman, Bill Biligetu, Yong-Bi Fu

**Affiliations:** 1 Department of Plant Sciences, College of Agriculture and Bioresources, University of Saskatchewan, Saskatoon, Saskatchewan, Canada; 2 Plant Gene Resources of Canada, Saskatoon Research and Development Centre, Agriculture and Agri-Food Canada, Saskatoon, Saskatchewan, Canada; Institute of Genetics and Developmental Biology Chinese Academy of Sciences, CHINA

## Abstract

Crested wheatgrass [*Agropyron cristatum* (L.) Gaertn.] provides high quality, highly palatable forage for early season grazing. Genetic improvement of crested wheatgrass has been challenged by its complex genome, outcrossing nature, long breeding cycle, and lack of informative molecular markers. Genomic selection (GS) has potential for improving traits of perennial forage species, and genotyping-by-sequencing (GBS) has enabled the development of genome-wide markers in non-model polyploid plants. An attempt was made to explore the utility of GBS and GS in crested wheatgrass breeding. Sequencing and phenotyping 325 genotypes representing 10 diverse breeding lines were performed. Bioinformatics analysis identified 827, 3,616, 14,090 and 46,136 single nucleotide polymorphism markers at 20%, 30%, 40% and 50% missing marker levels, respectively. Four GS models (BayesA, BayesB, BayesCπ, and rrBLUP) were examined for the accuracy of predicting nine agro-morphological and three nutritive value traits. Moderate accuracy (0.20 to 0.32) was obtained for the prediction of heading days, leaf width, plant height, clump diameter, tillers per plant and early spring vigor for genotypes evaluated at Saskatoon, Canada. Similar accuracy (0.29 to 0.35) was obtained for predicting fall regrowth and plant height for genotypes evaluated at Swift Current, Canada. The Bayesian models displayed similar or higher accuracy than rrBLUP. These findings show the feasibility of GS application for a non-model species to advance plant breeding.

## 1 Introduction

Crested wheatgrass [*Agropyron cristatum* (L.) Gaertn.] is a perennial, outcrossing, cool season grass native to Eurasia. It belongs to the genus *Agropyron*, and has three ploidy forms: diploids, tetraploids and hexaploids [1–4]. Crested wheatgrass has acclimatized in the Canadian prairies since its introduction in early 1900’s [1], and is an important perennial pasture grass in western Canadian grasslands occupying approximately 1.7 million ha mostly in Alberta and Saskatchewan [5, 6]. It is highly valued for early spring growth, high palatability, nutritive value and yield [7–9]. It is also valued for its drought tolerance, and winter hardiness due to its extensive fibrous root system [7, 8]. Its persistence and competitiveness have continued to provide higher yields than native range species even several decades after seeding, irrespective of heavy grazing and trampling [7, 8, 10]. Crested wheatgrass is also known to possess resistance to diseases, and tolerance to abiotic stress, which are important to, and have been utilized in, wheat (*Triticum aestivum*), and barley (*Hordeum vulgare*) breeding [11–14]. High palatability and high nutrient content in crested wheatgrass are short lived during the active growth period and decline rapidly after plant heading. Thus, developing later maturing crested wheatgrass cultivars is valuable to maintain yield and nutritive value into the summer grazing season.

Genetic variation within and among crested wheatgrass populations is high, offering ample possibility of genetic improvement [15]. Breeding and improvement in this species is based on recurrent phenotypic selection alone or in combination with pedigree information, to assess the breeding values of individuals [16–18]. Many such traits are quantitative in nature and under the influence of many genes with small effects. Population improvement involving such traits is carried out with trait evaluation in replicated trials under different environments. Varietal improvement of crested wheatgrass through phenotypic evaluation and selection is a long process, often requiring 10–15 years, leading to a slow rate of genetic gain. The rate of genetic gain in forage species is < 1% per year [16, 19], which is lower than that of major cereal crop species. Several factors such as a highly outcrossing nature due to self-incompatibility, prevalence of high levels of genetic heterozygosity and heterogeneity within crested wheatgrass species [15], genotypic and environmental effects and their interactions are bottlenecks towards accelerated breeding of crested wheatgrass. Thus, it is difficult to perform trait evaluation through current methods of phenotypic evaluation alone. Most importantly, lack of an effective marker system for marker-assisted breeding is another major constraint. In addition, highly heterozygous individuals and the heterogeneous nature of populations, coupled with the genetic complexity of traits, can further limit the application of marker assisted selection. Thus, exploring novel breeding strategies such as genomic selection (GS) that associates DNA marker variation to phenotypic variation with statistical models is desired for accelerated breeding of this perennial species.

Availability of molecular markers for non-model polyploid plant species like crested wheatgrass is a recent development. Next generation sequencing technologies have offered genome-wide markers for crops with no prior sequence information. Genotyping-by-sequencing (GBS) is a powerful genomic approach for identification of genome-wide single nucleotide polymorphisms (SNPs) of non-model plants [20–24]. This approach generates high-density genotypic information at reduced cost without requiring a reference genome sequence [24]. Peterson et al. [23] and Baral et al. [15] have described the detailed GBS approach. The sample-by-variant matrix obtained was used for genomic selection application. Irrespective of the robustness of the GBS application, there are many missing data points, uneven genome coverage, complex bioinformatics, and issues related to polyploidy, limiting its application [25–27]. These drawbacks can be overcome with a GBS-based pipeline, called Haplotag [28], which can generate tag-level haplotype and SNP data for polyploid organisms [15].

Early marker assisted selection (MAS) was largely based on limited number of DNA markers and their association with quantitative trait loci (QTL) and could out-perform phenotypic selection alone [29]. However, weak association between the markers and traits across different genetic backgrounds, and the small proportion of genetic variation explained by the small numbers of markers used to trace major QTLs, limits its application [30, 31]. Genomic selection overcomes some limitations of MAS [30]. Using the genome-wide SNP markers, the ability to estimate marker effects for a quantitative trait is enhanced, so the prediction of individual breeding value (genomic estimated breeding value, GEBV) would be more reliable [30, 32], and more accurate parental selection can be obtained [33]. Simulations and empirical studies have confirmed that GS can significantly accelerate breeding programs, and improve genetic gain compared to phenotypic selection or QTL approaches [32, 34]. The potential of GS has been recently described for forage crop breeding and has been demonstrated in alfalfa (*Medicago sativa* L.) [35–37], intermediate wheatgrass (*Thinopyrum intermedium* L.) [38], switchgrass (*Panicum virgatum* L.) [39–42], and perennial ryegrass (*Lolium perenne* L.) [43–45]. However, little is known about the feasibility of GS application in crested wheatgrass breeding. So far, utilization of available marker systems in crested wheatgrass has been limited to the study of the genetic relationship of breeding lines, ecotypes and species of crested wheatgrass [6, 15, 46], linkage mapping [47–49] and identification of flowering time related and differentially expressed genes [9, 50].

This study was conducted with the objectives: (1) to apply GBS in combination with the Universal Network Enabled Analysis Kit (UNEAK) and the Haplotag pipelines to identify genome-wide SNP markers and; (2) to assess the feasibility of GS for complex traits in diploid and tetraploid crested wheatgrass lines.

## 2 Materials and methods

I confirm that the University of Saskatchewan and Agriculture and Agri-Food Canada ethics committees approved this study. We don't issue field permit. This is a part of our regular research study.

### 2.1 Plant materials

The study material comprised ten lines of crested wheatgrass (five cultivars: Fairway, Kirk, AC-Goliath, AC-Parkland and NewKirk, and five breeding lines: S8959E, S9491, S9516, S9542 and S9556) (Table 1). AC Parkland, Fairway and S9542 were diploid cultivars, while the other seven lines were tetraploids. These lines were made available from the forage breeding program of the University of Saskatchewan. Two field trials were established at the Agriculture and Agri-Food Canada (AAFC) Saskatoon Research Farm, Saskatoon, SK and Swift Current Research and Development Center, Swift Current, SK in 2014 using randomized complete block designs (RCBD) with four replications. Each line had 16 genotypes per replication. Each genotype was spaced 1m within and between rows. Data on agro-morphological and nutritive value traits were collected for two years in 2015 and 2016. For the marker data generation, young leaf tissues were collected from 160 genotypes (16 randomly selected genotypes for each of the 10 lines in four replications) from Saskatoon and 165 genotypes (16 randomly selected genotypes for each of the 10 lines in four replications and additional randomly selected 5 genotypes) from Swift Current and stored at -80°C prior to DNA extraction. A total of 325 genotypes from the 10 lines were used for bioinformatics analysis.

**Table 1:** List of the 10 crested wheatgrass (*A*. *cristatum*) lines used in the study.

Lines	Origin	Type	Ploidy
Fairway	Canada	Cultivar	Diploid
Kirk	Canada	Cultivar	Tetraploid
AC-Goliath	Canada	Cultivar	Tetraploid
AC-Parkland	Canada	Cultivar	Diploid
NewKirk	Canada	Cultivar	Tetraploid
S8959E	Siberia/Canada	Breeding line	Tetraploid
S9491	Canada	Breeding line	Tetraploid
S9516	Canada	Breeding line	Tetraploid
S9542	Canada	Breeding line	Diploid
S9556	Canada	Breeding line	Tetraploid

### 2.2 Agro-morphological traits

The agro-morphological traits evaluated for the 160 genotypes in Saskatoon and 165 genotypes in Swift Current included early spring vigor score (ESV), heading days (DTH), plant height (PH), leaf width (LW), clump diameter (CD), dry matter yield (DMY), regrowth score after harvest (RGAH) and fall regrowth score (FRG). Tillers per plant (TPP) was evaluated at the Saskatoon site. Descriptions of the measurement of these agro-morphological traits are presented in Table 2. DTH were expressed as growing degree days (GDD) [39] using a base temperature of 0°C.

**Table 2:** Description of the measurement of agro-morphological traits.

Traits	Trait description	Year
Early spring vigor (ESV)	1 = least vigorous; 5 = most vigorous, visually scored on first week of May.	2015–2016
Heading days (DTH)	50% of stems have 50% emerged panicles.	2015–2016
Plant height (PH)	Height measured in centimeter from base of the stem to tip of the panicle.	2015–2016
Leaf width (mm) (LW)	Widest part of penultimate leaf measured in millimeter.	2015–2016
Clump diameter (CD)	Measured on clump after harvest in centimeter.	2015–2016
Tillers per plant (TPP)	Number of tillers in each genotype.	2015–2016
Dry matter yield (DMY)	Each genotype harvested were dried for 48h at 60 °C in a forced air oven and weighed in gram, harvesting done on last week of July.	2015–2016
Regrowth score after harvest (RGAH)	1 = least vigorous; 5 = most vigorous, visually scored on last week of August.	2015–2016
Fall regrowth score (FRG)	1 = least vigorous; 5 = most vigorous, visually scored on first week of October.	2015–2016

### 2.3 Nutritive value traits

The genotypes were sub-sampled after dry matter determination during the growing seasons of 2015 and 2016 for forage nutritive value determination. The sub-samples were ground through a 1-mm screen Wiley mill (Thomas-Wiley, Philadelphia, PA). The ground samples were stored in plastic bags prior to determination of crude protein (CP), neutral detergent fiber (NDF) and acid detergent fiber (ADF). Nitrogen concentration was determined by the Dumas combustion method using the Leco CN 628 Dumas analyzer (Leco Corporation, St. Joseph, MI) for all the Saskatoon samples and the 2015 Swift Current samples, while the Kjeldahl method was used for samples from Swift Current in the year 2016. Then, nitrogen content was converted to CP multiplying by a conversion factor of 6.25. NDF and ADF concentrations were determined using an automated Ankom^2000^ fiber analyzer (ANKOM Technology Corporation, New York, USA) following manufacturer’s instructions.

### 2.4 Genotyping-by-sequencing

For each of the 325 genotypes, protocols of NucleoSpin^®^ Plant II Kit (Macherey-Nagel, Bethlehem, PA, USA) were used to extract DNA from 0.1 g finely ground tissue and was eluted in a 1.5 mL Eppendorf tube with Elution Buffer. DNA quality was measured by comparing the absorptions at 260 and 280 nm using NanoDrop 8000 (Thermo Fisher Scientific, Waltham, MT, USA). Further quantification of the DNA samples was through the Quant-iTTM PicoGreen^®^ dsDNA assay kit (Invitrogen, Carisbad, CA, USA) and final dilution to 60 ng/μl with 1× TE buffer was done before sequencing analysis.

A genetic diversity-focused GBS (gd-GBS) protocol was used for the preparation of multiplexed GBS libraries [15, 23]. Briefly, restriction enzyme combinations *Pst*I and *Msp*I (New England Biolabs, Whitby, ON, Canada) digested 200 ng of purified genomic DNA in each library. On to the 5′ and 3′ ends of the restriction fragments, ligation of customized adapters by T4 ligase was carried out. Then, AMPure XP kit (Beckman Coulter, Brea, CA, USA) was used for purification of the ligated fragments. Through PCR amplification, Illumina TruSeq HT multiplexing primers were added following the purification. The amplicon fragments were further quantified, concentrated, and pooled to form 4 subgroups of 12 samples each. Using a Pippin Prep instrument (Sage Science, Beverly, MA, USA), pre-selection of the samples in the subgroups for an insert size range of 100–400 bp were done before pooling the samples into a library. Each pooled library was diluted to 6 pM, and denatured with 5% of sequencing-ready Illumina PhiX Library Control (Illumina, San Diego, CA, USA) that can serve for calibration. Sequencing was completed using an Illumina Hiseq2500 Instrument with paired-ends of 125 bp in length. HiSeq runs of 6 libraries generated 672 FASTQ sequence files from 336 genotypes (including randomly selected 11 technical replicates) of 10 lines (one forward and one reverse for each of 336 genotypes). All FASTQ files were deposited to Sequence Read Archive (SRA) database under NCBI with SRA accession PRJNA599212 and submission ID SUB6703766 (https://www.ncbi.nlm.nih.gov/sra/PRJNA599212).

### 2.5 Bioinformatics analysis

Bioinformatics analysis began with sequence (FASTQ) data cleaning, using Trimmomatic version 0.36 [51] to remove any sequenced-through Illumina adapters, low quality sequences (sliding window of 10 bases, average Phred of 20), and fragments under 64 bases long. As the UNEAK-GBS pipeline [52] only considers sequences of 64 bp (after barcode removal) with an intact 5-base *Pst*I residue (TGCAG) at the beginning, each FASTQ file of 125 bp was split with a custom Perl script *fastqHiseqCutandCode-Pst*.*pl* available via Figshare (10.6084/m9.figshare.11530677, S1 Text) to get the first 64 bases with the *Pst*I residual restriction site. The script also provided an arbitrary barcode sequence (CATCAT) at the start of each sequence fragment, since the UNEAK pipeline expects to de-convolute barcoded sequence reads which are not already separated by sample. The 70-base-long fragments formed, thereafter, were recognized by the UNEAK-GBS pipeline [52], and passed into UNEAK.

The fragment set (70 bases long) was analyzed with UNEAK and the Haplotag pipeline [28], resulting in the analysis of a total of 59 bases of genetic sequence. S2 Text, Section B available in Figshare (10.6084/m9.figshare.11530677) describes the procedures to run UNEAK. Two types of meta data files, a single mergedAll.txt (all tags observed more than 10 times) provided as S3 Text in Figshare (10.6084/m9.figshare.11530677) and a set of individual tagCount files (one per sample) needed for the Haplotag pipeline were generated from the UNEAK run.

Haplotag was run with the parameters and filtering threshold settings described in the HTinput.txt file available via Figshare (10.6084/m9.figshare.11530677, S4 Text) and generated a matrix of samples by SNP loci as described in S2 Text, Section B. A set of tag-level haplotypes (“HTgenos”) are first generated by Haplotag, followed by a set of SNP data derived from these haplotypes (“HTSNPgenos”). These two data types are technically redundant, so choosing one of them relies on the implementation and preference of software. In the present study, most (97.5%) haplotypes were found to contain only a single SNP; thus, we decided to analyze the SNP dataset for simplicity and compatibility with downstream analysis software.

The character by Taxa (CbyT) program supplied by N. Tinker [28] was used to generate a filtered SNP file. In brief, Haplotag generated “HTSNPGenos” file, which was run with CbyT. The “minimum presence” value in CbyT was set to 80%, 70%, 60%, and 50% for 20%, 30%, 40%, and 50% missing data, respectively. A SNP-by-sample matrix in the output files available via Figshare (10.6084/m9.figshare.11530677, S5–S8 Text) was used in further analyses. Additionally, S1–S4 Files and S9–S15 Text available via Figshare (10.6084/m9.figshare.11530677) provide descriptions of the genotypes, phenotypes, the batch files, and custom Perl and Shell scripts used. Analyses from FASTQ file separation to SNP generation were conducted using Microsoft Windows 7 64-bit OS with an Intel (R) Xeon (R) CPU E5-2623 v3 @ 3.00 GHz (8 threads) and 32 GB RAM.

### 2.6 GBS SNP data imputation and filtering

The SNP marker information for the missing markers at each level (20%, 30%, 40% and 50%) were reconstructed using a probabilistic Principal Component Analysis (probabilistic PCA) that integrates expectation maximization approach with probabilistic models using the R package “pcaMethods” [53, 54]. Following the imputation, SNPs were filtered using the technical replicates to get the same SNP information at each locus in the original and the replicate. Later, allele frequency of these SNPs was calculated independently for the 160 genotypes for nine agro-morphological and three nutritive value traits in Saskatoon, and 159 genotypes for eight agro-morphological traits and 156 genotypes for three nutritive value traits in Swift Current, respectively. The number of genotypes for agro-morphological and nutritive value traits evaluated at Swift Current differed as the genotypes with missing phenotypic records in one or both the years were excluded from further analysis. Within each of these subsets, SNPs with minor allele frequency (MAF) < 0.05 were removed prior to their use in genomic selection models.

### 2.7 Genetic structure analysis

Our study materials consisted of diploid and tetraploid crested wheatgrass lines. Thus, it was important to determine the existence of genetic structure in the study material and estimate the effect of SNPs arising from the genetic structure prior to genomic selection analysis. For this, a principal coordinates analysis (PCoA) was conducted using the R routine AveDissR [55] to assess genotypic associations of the assayed samples. Plots of the first two resulting principal coordinates were generated. Based on the resulting genotypic associations, we reasoned that the first two PCoAs of the imputed markers (filtered with technical replicates but without filtering for MAF < 0.05) at each missing level would sufficiently account for these genetic differences due to ploidy. Hence, we fitted a model of Best Linear Unbiased Predictor (BLUP) for each trait where trait was the response variable and the first two PCoAs were the explanatory variables. The residuals obtained from each model fitting were used for genomic selection.

### 2.8 Phenotypic evaluation

Outliers for each of the phenotypic traits at each location were examined using studentized deleted residuals [56] from a mixed linear model including year and line as random effects in SAS 9.4 [57]. Then, the Box-cox procedure was implemented to determine the optimal transformation of the traits [58]. Best linear unbiased predictors were estimated for each trait in each genotype across year using a mixed linear model with genotype and year as random effects fitted in R using the package “lme4” [59]. Variance component estimates from the model used to obtain the BLUPs were applied to estimate broad sense heritability (repeatability) on a genotype mean basis ($\hat{H}$) [60, 61].

### 2.9 Genomic selection using SNP data

To assess the applicability of genome-wide markers to predict phenotypes of agro-morphological and nutritive value traits in crested wheatgrass, four additive genomic selection models, including BayesA [30], BayesB [30], BayesCπ [62], and Ridge Regression Best Linear Unbiased Predictor (rrBLUP) [30] were chosen. These models differ in their assumptions of the marker effects. BayesA assumes each marker has a distinct variance such that there are many markers with small effects and few markers with moderate effects. BayesB assumes only a portion of the markers explain total variance and most markers explain zero variance [30]. BayesCπ assumes a common marker effect variance and allows some markers to have no effect [62]. rrBLUP assumes that all markers have common variance with small but non-zero effect and thus shrinks equally for each marker effect [30]. All statistical modeling was done in R 3.5.1 [63]. The rrBLUP model was implemented using “rr-BLUP” package [64] while Bayesian models were implemented using the “BGLR” package [65]. The model parameters were considered following the package instructions.

The prediction accuracy of the models was assessed by splitting the data into a training set and a validation set and repeated 500 times. The genotypes were randomly partitioned into two equally sized subgroups. Then, for each repeat, the randomly sampled half of the subgroups was used as the training set and the remaining half as the validation set. The validation set was used to assess the correlation between observed and predicted trait values (GEBVs). For GS model comparison, the same training and validation sets were used for all four GS models. Prediction accuracy of each model for each trait was the average Pearson correlation coefficient across the 500 repeats. A nonparametric ANOVA with two factors was considered to assess the effects of GS model and location, as the estimates in this study were not always normally distributed, and the R “ARTool” package [66] was applied for each trait, where prediction accuracy of each trait at each repeat was considered as response. Pairwise comparison was made for the prediction accuracies of GS models by each location using the R “emmeans” package [67].

## 3 Results

### 3.1 Phenotypic variation

There was substantial variation in each of the agro-morphological and nutritive value traits (Tables 3 and 4). In Saskatoon, the difference between minimum and maximum values ranged from 1.3-fold for NDF to 13-fold for TPP (Table 3). The average broad-sense heritability for the agro-morphological traits was 0.57 with a range of 0.38 (ESV) to 0.73 (TPP). For nutritive value, broad-sense heritability ranged from 0.27 (CP) to 0.54 (ADF). In Swift Current, the difference between minimum and maximum values ranged from 1.2-fold for DTH to 9-fold for DMY (Table 4). Heritability among the agro-morphological traits ranged from 0.31 (FRG) to 0.74 (PH) with an average heritability of 0.53. Heritability among the nutritive value traits ranged from 0.28 (CP) to 0.58 (ADF).

**Table 3:** Statistics for the distributions of nine agro-morphological and three nutritive value traits, and estimated broad-sense heritability on genotype-mean basis for crested wheatgrass evaluated in two summers at Saskatoon, Canada.

Traits	Unit	No. genotype	Year	Mean	Standard deviation	Range	Heritability (H^2^)
Early spring vigor (ESV)		160	2015	3.7	0.9	1.0–5.0	0.38
			2016	4.28	0.9	2.0–5.0	
Heading days (DTH)	GDD	160	2015	703.0	63.7	543.2–864.5	0.36
			2016	708.7	82.0	543.8–925.0	
Plant height (PH)	cm	160	2015	82.2	13.9	30.0–118.0	0.72
			2016	97.9	16.7	35.0–133.0	
Tillers per plant (TPP)		160	2015	133.4	53.4	33.0–290.0	0.73
			2016	379.7	135.8	64.0–833.0	
Leaf width (LW)	mm	160	2015	9.3	1.6	4.0–13.7	0.62
			2016	7.3	1.4	4.0–11.0	
Clump diameter (CD)	cm	160	2015	18.8	3.8	8.0–29.0	0.63
			2016	22.1	3.6	14.0–30.0	
Regrowth score (RGAH)		160	2015	3.8	0.9	1.0–5.0	0.55
			2016	3.5	1.2	1.0–5.0	
Dry matter yield (DMY)	g	160	2015	258.8	78.5	70.8–504.9	0.67
			2016	417.4	109.8	71.7–709.7	
Fall regrowth score (FRG)		160	2015	2.9	1.1	1.0–5.0	0.51
			2016	3.8	1.0	1.0–5.0	
Acid detergent fiber (ADF)	%	160	2015	36.6	3.6	24.1–46.3	0.54
			2016	37.3	2.9	31.3–45.8	
Neutral detergent fiber (NDF)	%	160	2015	59.5	3.9	49.6–69.9	0.42
			2016	59.9	3.2	50.9–68.6	
Crude protein (CP)	%	160	2015	4.4	1.1	2.1–7.9	0.27
			2016	3.2	0.8	2.0–6.4	

**Table 4:** Statistics for the distributions of eight agro-morphological and three nutritive values traits, and estimated broad-sense heritability on genotype-mean basis for crested wheatgrass evaluated in two summers at Swift Current, Canada.

Traits	Unit	No. genotypes	Year	Mean	Standard deviation	Range	Heritability (H^2^)
Early spring vigor (ESV)		159	2015	3.8	1.0	1.0–5.0	0.43
			2016	3.9	0.7	2.0–5.0	
Heading days (DTH)	GDD	159	2015	683.6	44.5	648.8–819.9	0.39
			2016	748.3	70.0	698.3–845.7	
Plant height (PH)	cm	159	2015	85.5	12.6	43.8–114.2	0.74
			2016	81.1	13.9	46.8–110.7	
Leaf width (LW)	mm	159	2015	9.0	1.6	5.8–13.2	0.65
			2016	6.8	1.2	4.2–10.80	
Clump diameter (CD)	cm	159	2015	17.3	2.7	11.0–25.0	0.62
			2016	28.1	3.8	16.0–37.0	
Regrowth score (RGAH)		159	2015	3.7	1.1	1.0–5.0	0.48
			2016	4.1	0.7	1.0–5.0	
Dry matter yield (DMY)	g	159	2015	492.5	128.2	106.7–829.8	0.60
			2016	525.5	153.6	122.0–1104.0	
Fall regrowth score (FRG)		159	2015	3.4	0.7	2.0–5.0	0.31
			2016	4.2	0.6	2.0–5.0	
Acid detergent fiber (ADF)	%	156	2015	33.8	4.0	24.7–48.6	0.58
			2016	35.4	3.0	27.5–46.9	
Neutral detergent fiber (NDF)	%	156	2015	56.6	4.6	46.6–69.0	0.45
			2016	61.8	3.9	51.8–77.6	
Crude protein (CP)	%	156	2015	4.6	0.9	2.6–7.3	0.28
			2016	9.4	2.1	4.6–14.9	

### 3.2 Genotyping-by-sequencing for SNP discovery

The HiSeq run of 336 genotypes from the 10 crested wheatgrass lines (Table 1) yielded approximately 888.2 million raw forward sequence reads from six libraries. The number of raw forward sequence reads per sample ranged from 677,492 to 5,578,827 with an average of 2,643,717. Combined UNEAK and Haplotag analysis at the 20%, 30%, 40%, and 50% levels of missing data generated 827, 3,616, 14,090, and 46,136 SNPs, respectively, across the 336 genotypes (Table 5 and S1 File available via Figshare (10.6084/m9.figshare.11530677). In addition, this analysis also generated many meta-genomic files associated with the SNP discovery, which are described and accessible via Figshare (10.6084/m9.figshare.11530677, S2 Text). The data filtering done by removal of SNPs differing with technical replicates and again with SNPs having MAF <0.05 resulted in 286, 1,206, 5,437 and 17,003 SNPs at 20%, 30%, 40%, and 50% levels of missing data in Saskatoon, respectively (Table 5). Likewise, 257, 1,154, 5,321 and 16,771 SNPs were found for agro-morphological traits and 255, 1,142, 5,278 and 16,692 SNPs for nutritive value traits at 20%, 30%, 40%, and 50% levels of missing data respectively in Swift Current (Table 5). The number of genotypes available for evaluation varied in Swift Current for agro-morphological (159 genotypes) and nutritive value traits (156 genotypes) resulting in the difference in the final SNPs counts after filtering.

**Table 5:** Counts of SNPs at four levels of missing data and different filtering criteria.

Missing level	SNP Counts	Average missing rate of SNPs (%)	SNPs filtered with technical replicates	SNPs with MAF^a^ ≥ 0.05 Saskatoon	SNPs with MAF ≥0.05 agro-morphological traits, Swift Current	SNPs with MAF ≥0.05 nutritive value traits, Swift Current
20%	827	15.01	659	286	257	255
30%	3616	23.51	2149	1206	1154	1142
40%	14,090	32.79	8091	5437	5321	5278
50%	46,136	41.95	21,957	17,003	16,771	16,692

^a^ MAF, minor allele frequency.

### 3.3 Genetic structure of assayed samples

**Fig 1:**
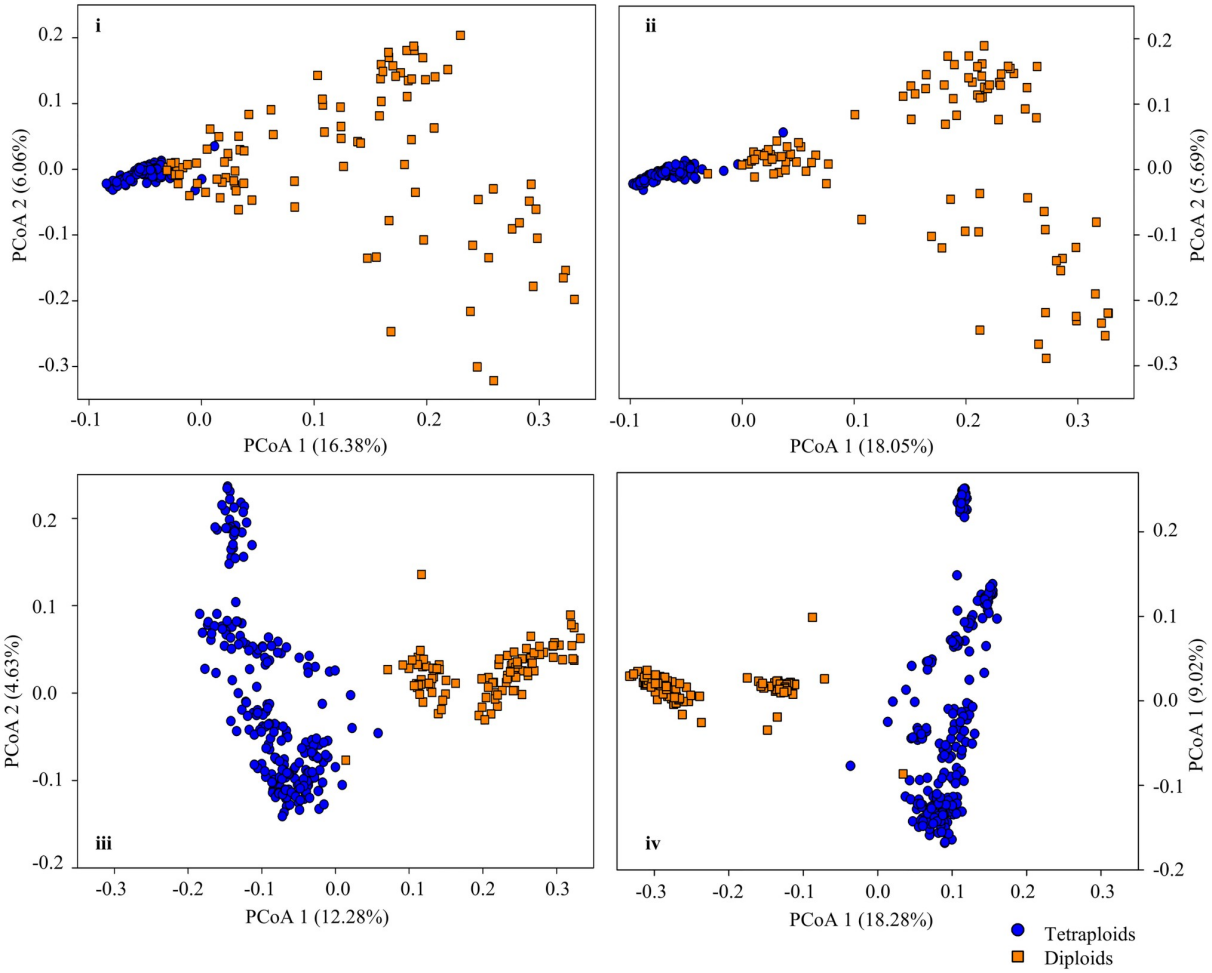
Genetic associations of diploid and tetraploid crested wheatgrass genotypes used for the genomic selection study as explained by PCoA1 and PCoA2 of principal coordinate analysis. (i) SNPs at 20% missing level; (ii) SNPs at 30% missing level; (iii) SNPs at 40% missing level; and (iv) SNPs at 50% missing level.

Investigation of the genetic structure with PCoA revealed that diploid lines clustered separately from the tetraploid cultivars and breeding lines with little overlap (Fig 1). This suggests the first two PCoAs of the SNPs were able to infer a sufficient level of genetic discrepancy between the two ploidy levels and support our hypothesis of the effectiveness of using the first two PCoAs to mitigate the SNP effect arising from the population structure prior to a genomic selection analysis.

### 3.4 Accuracy of genomic prediction in crested wheatgrass

**Fig 2:**
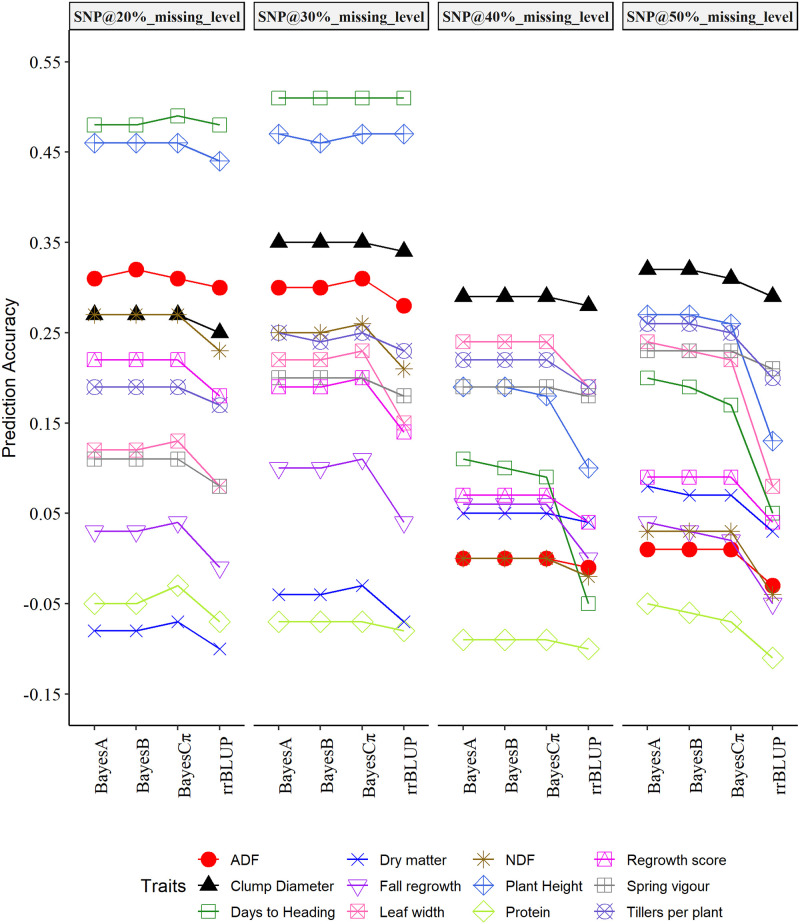
Prediction accuracy of four genomic selection models for genotypes evaluated at Saskatoon, Canada.

**Fig 3:**
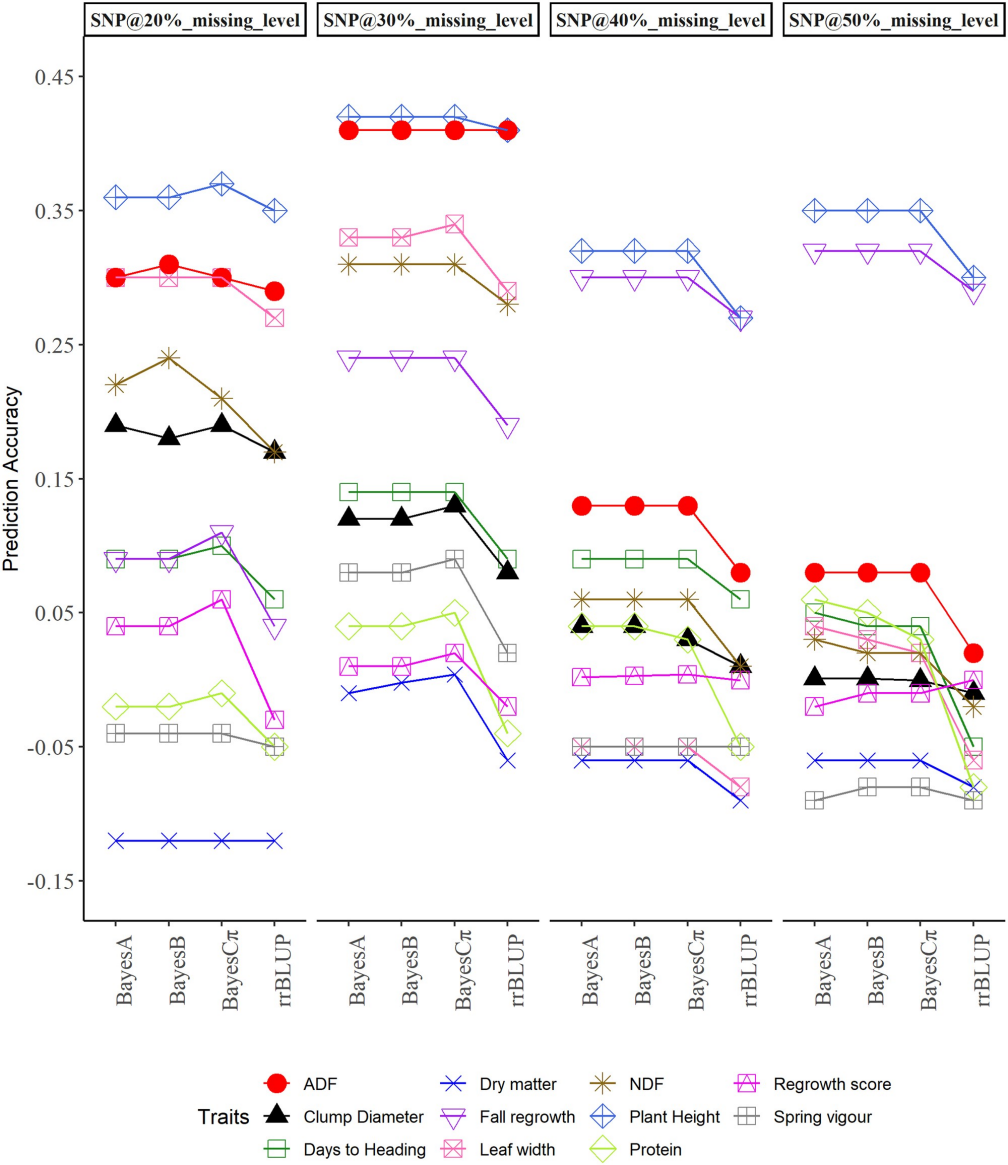
Prediction accuracy of four genomic selection models for genotypes evaluated at Swift Current, Canada.

The average prediction accuracy of the four genomic selection models evaluated for crested wheatgrass varied for each trait at the four SNP densities corresponding to the missing marker levels (20%, 30%, 40% and 50%) of SNP markers (Table 6 and Figs 2 and 3). However, the prediction accuracy of the GS models except rrBLUP were similar for most of the traits. The genomic selection models were implemented separately for genotypes evaluated in Saskatoon and Swift Current. In Saskatoon, the average prediction accuracy of GS models at the 50% missing level of SNP information ranged from low to moderate prediction abilities (0.030 to 0.319) for DMY, DTH, LW, PH, CD, TPP. The prediction accuracy of ESV, RGAH and FRG ranged from -0.054 to 0.231. While, for ADF, NDF and CP, prediction accuracy ranged from -0.108 to 0.032 (Table 6). In Swift Current, the average prediction accuracy of GS models at 50% missing level of SNPs information ranged from -0.077 to 0.350 for DMY, DTH, LW, PH, and CD. The prediction accuracy of ESV, RGAH and FRG ranged from -0.088 to 0.323; while, for ADF, NDF and CP prediction accuracy ranged from -0.079 to 0.084 (Table 6).

**Table 6:** Prediction accuracies of four genomic selection models at 50% missing level of SNPs information for agro-morphological and nutritive value traits of crested wheatgrass evaluated at Saskatoon and Swift Current, Canada.

Models	Saskatoon				Swift Current			
Traits/Location	BayesA	BayesB	BayesCπ	rrBLUP	BayesA	BayesB	BayesCπ	rrBLUP
Early spring vigor	0.231^a^ (0.004)	0.230^a^ (0.004)	0.228^a^ (0.004)	0.206^b^ (0.004)	-0.086^a^ (0.003)^a^	-0.084^a^ (0.003)	-0.084^a^ (0.003)	-0.088^a^ (0.003)
Heading days	0.196^a^ (0.004)	0.187^a^ (0.004)	0.170^b^ (0.004)	0.053^c^ (0.004)	0.049^a^ (0.004)	0.045^a^ (0.004)	0.036^a^ (0.004)	-0.047^b^ (0.004)
Leaf width	0.240^a^ (0.004)	0.234^ab^ (0.004)	0.221^b^ (0.004)	0.082^c^ (0.004)	0.036^a^ (0.004)	0.032^ab^ (0.004)	0.020^b^ (0.004)	-0.061^c^ (0.003)
Plant height	0.274^a^ (0.004)	0.268^ab^ (0.004)	0.256^b^ (0.004)	0.126^c^ (0.005)	0.350^a^ (0.003)	0.348^a^ (0.003)	0.345^a^ (0.003)	0.300^b^ (0.004)
Clump diameter	0.319^a^ (0.003)	0.316^a^ (0.004)	0.310^a^ (0.004)	0.291^b^ (0.003)	0.001^a^ (0.004)	0.001^a^ (0.004)	0.001^a^ (0.004)	-0.006^a^ (0.004)
Regrowth score	0.093^a^ (0.004)	0.090^a^ (0.004)	0.088^a^ (0.004)	0.039^b^ (0.004)	-0.016^b^ (0.004)	-0.015^b^ (0.004)	-0.007^ab^ (0.004)	0.000^a^ (0.004)
Dry matter yield	0.076^a^ (0.004)	0.073^a^ (0.004)	0.069^a^ (0.004)	0.030^b^ (0.004)	-0.055^a^ (0.004)	-0.058^a^ (0.004)	-0.063^a^ (0.004)	-0.077^b^ (0.003)
Fall regrowth	0.043^a^ (0.004)	0.031^ab^ (0.004)	0.018^b^ (0.004)	-0.054^c^ (0.004)	0.323^a^ (0.004)	0.320^a^ (0.004)	0.318^a^ (0.004)	0.291^b^ (0.003)
Acid detergent fiber	0.013^a^ (0.004)	0.014^a^ (0.004)	0.009^a^ (0.004)	-0.033^b^ (0.004)	0.084^a^ (0.004)	0.082^a^ (0.004)	0.078^a^ (0.004)	0.020^b^ (0.004)
Neutral detergent fiber	0.032^a^ (0.004)	0.028^a^ (0.004)	0.020^a^ (0.004)	-0.036^b^ (0.004)	0.030^a^ (0.004)	0.025^a^ (0.004)	0.020^a^ (0.004)	-0.022^b^ (0.003)
Crude protein	-0.053^a^ (0.004)	-0.060^a^ (0.004)	-0.072^b^ (0.004)	-0.108^c^ (0.003)	0.059^a^ (0.004)	0.052^a^ (0.004)	0.033^b^ (0.004)	-0.079^c^ (0.003)
Tillers per plant	0.260^a^ (0.004)	0.256^a^ (0.004)	0.250^a^ (0.004)	0.200^b^ (0.004)	-	-	-	-

Within each location the prediction accuracies followed by same letters are not significantly different at α = 0.05. The values in the parenthesis are standard errors.

Comparison of four additive GS models, BayesA, BayesB, BayesCπ and rrBLUP at a SNP density corresponding to 50% missing level for traits evaluated at Saskatoon exhibited similar prediction abilities of the Bayesian models for CD, DMY, ESV, TPP, RGAH, and ADF, while prediction accuracy of rrBLUP was the lowest (Table 6) for these traits. This is evident from average prediction accuracy of the models and associated standard errors (Table 6). Overall, prediction accuracy of rrBLUP was lower compared to the other three models for all of the evaluated traits. Prediction accuracy of Bayes A was always the highest or similar whereas, the prediction accuracy of BayesCπ was lower or similar to the remaining Bayesian models (Table 6). Similarly, the prediction accuracy of the four GS models at SNP density corresponding to 50% missing level for traits evaluated at Swift Current were similar for CD, ESV. The prediction accuracy of the Bayesian models were similar for DMY, PH, FRG, ADF, and NDF, whereas, prediction accuracy of rrBLUP was lower than the Bayesian models (Table 6). Overall, the prediction accuracy of rrBLUP was lower compared to BayesA, BayesB, and BayesCπ for all evaluated traits except ESV. Prediction accuracy of Bayes A was always highest or similar whereas, prediction accuracy of BayesCπ was always lower, or similar, to the remaining Bayesian models (Table 6). The prediction accuracies of the GS models were statistically significant except for two traits at Swift Current according to the pairwise comparison of the prediction accuracies for most of the traits (Table 6). The prediction accuracies were statistically significant for the GS models, location and their interactions for most of the traits as observed from the conservative nonparametric ANOVA (Table 7).

**Table 7:** Analysis of variance with genomic selection model and location as factors for 11 measured traits.

Agro-morphological trait						
Source	df	Dry matter yield	Clump diameter	Heading days	Leaf width	Plant height
Model	3	37.13***^a^	8.05***	434.85***	449.16***	296.04***
Location	1	2383.21***	11208.69***	2575.55***	5017.65***	1763.52***
Model × Location	3	5.1**	2.42	17.41***	27.40***	79.78***
Source	df	Early spring vigor	Fall regrowth score	Regrowth after harvest		
Model	3	9.10***	128.84***	15.54***		
Location	1	11176.05***	10647.93***	1230.37***		
Model × Location	3	4.68**	31.42***	41.00***		
Nutritive value trait						
Source	df	Acid detergent fiber	Neutral detergent fiber	Crude protein		
Model	3	105.16***	117.89***	324.09***		
Location	1	606.03***	0.86	1253.39***		
Model × Location	3	2.85*	2.73*	67.88***		

^a^ Significance level with *** for P≤0.001; ** for P≤0.01; and * for P≤0.05.

Our results demonstrated prediction accuracy of the GS models varied for each trait at each level of SNP density (corresponding to missing SNPs levels) at each location. Overall, in Saskatoon, the prediction accuracy improved with increase in SNP densities for DMY and LW. An increase in prediction accuracy with increasing SNP density from 20 to 30% missing levels, then a decrease at the 40% missing level, followed by an increase at the 50% missing level was observed for DTH, PH, CD,TPP and ESV. While, for RGAH, ADF, NDF, and CP, prediction accuracy of the models decreased with increasing SNP densities up to the 40% missing level and then improved with increasing SNP densities at 50% missing level except for rrBLUP. The prediction accuracy of the models for FRG increased up to 30% missing level and later decreased with increasing SNP densities up to 50% missing levels (Fig 2). Evaluation of each prediction model at each level of SNP density revealed higher prediction accuracy of BayesCπ at lower SNP densities (20 and/or 30% missing levels) for DMY, DTH, LW, RGAH, FRG, ADF, NDF and CP. Whereas, lower prediction accuracy of BayesCπ at higher SNP density (50% missing level) was observed for DTH, LW, PH, CD, TPP and FRG. The prediction accuracy of rrBLUP was the lowest compared to the other three models except for similar prediction accuracy as Bayesian models for DTH at 20 and 30% missing levels and BayesA, and BayesCπ for PH at 30% missing level (Fig 2).

Similarly, for the traits evaluated at Swift Current, an increase in the prediction accuracy with increasing SNP density was observed for FRG and CP. An increase in prediction accuracy with increasing SNP density from 20 to 30% missing level, then a decrease at 40% missing level, followed by an increase with increasing SNP density at the 50% missing level was observed for LW and PH. Increase in prediction accuracy with increasing SNP density from 20 to 30% missing level followed by a decrease with increasing SNP density up to 50% missing level was observed for DMY, DTH, ESV, ADF and NDF. Decreasing prediction accuracy with increasing SNP density was observed for CD and RGAH (Fig 3). Evaluation of each prediction model at each level of SNP densities revealed a higher prediction accuracy of BayesCπ at lower SNP densities (20 and/or 30% missing levels) for DMY, DTH, LW, PH, CD, ESV, RGAH, FRG, and CP at Swift Current. A higher prediction accuracy of BayesA at higher SNP density was observed for DTH, LW, NDF and CP. Likewise, BayesB and BayesCπ were higher in prediction accuracy at higher SNP density for ESV. Bayesian models were similar in prediction accuracy at higher SNP densities for DMY, PH, CD, FRG and ADF. The prediction accuracy of rrBLUP was the lowest except for higher prediction accuracy at the 50% missing level for RGAH. The prediction accuracy of rrBLUP was similar to that of Bayesian models for DMY, ESV and ADF at 20, 30 and 40% missing levels, respectively (Fig 3).

## 4 Discussion

This study, for the first time, investigated the prediction accuracy of genomic selection models in crested wheatgrass breeding utilizing the gd-GBS application for SNP marker generation. This study assessed and compared the prediction accuracy of four GS models at four levels of SNP densities. Overall, the GS models varied in prediction accuracy of the evaluated traits at each level of SNP density at each location. The findings revealed Bayesian models provided higher prediction accuracies than rrBLUP for the agro-morphological and nutritive value traits in crested wheatgrass. The traits with unknown genetic architecture in crested wheatgrass might have been under the influence of certain QTLs rather than a large number of QTLs, which would result for the higher prediction accuracies of Baysesian models compared to rrBLUP as rrBLUP assumes that all markers have common variance with small but non-zero effect [30]. The relationship between the prediction accuracy of the models, traits evaluated and the density of the SNP markers showed different trends with increased SNP densities. These findings show the feasibility of the GBS-based GS application in crested wheatgrass breeding, even for quantitative traits such as DMY.

Given the long breeding cycles required for varietal development in crested wheatgrass, genomic selection could significantly reduce the time for each breeding cycle by estimation of GEBVs at the seedling stages without phenotyping over multiple site-years. The prediction accuracies for the agro-morphological traits and the three forage nutritive value traits in our study are comparable to findings in other perennial forage crops [37, 39]. Moderate prediction accuracies (in the range of 0.20–0.35) for traits such as DTH, LW, PH, CD, ESV, FRG and TPP indicate the possibility of the application of genomic selection in crested wheatgrass to improve the genetic gain per unit of time for these traits. In studies on other plant species, genetic gain per year with GS was greater than MAS for the traits with prediction accuracies of 0.2 in maize (*Zea mays*) and 0.3 in winter wheat [68].

The present study also showed certain negative prediction values ranging from -0.11 to -0.01, for DMY, DTH, LW, CD, ESV, RGAH, FRG, ADF, NDF, and CP, in particular, at Swift Current site. Negative prediction values have also been reported from genomic selection studies in maize [68, 69], sugar beet (*Beta vulgaris* L.) [70], perennial ryegrass [44, 45] and switchgrass [39–41]. These negative predictions could have resulted from opposite linkage phases between markers and QTLs in training and prediction sets as discussed in other studies [70]. In this study, genotypes in the training and validation sets were half-sibs from different families selected for different traits, which could result in opposite linkage phase among the genotypes of training and validation sets. A previous simulation study suggested that more accurate predictions are obtained with the inclusion of multiple populations that have marker-QTLs in the same LD phase in the training set [71]. Low to negative prediction accuracies were found for biomass across growing seasons and years in a GS study of perennial ryegrass, which was suggested to be caused by environmental distinctness, prevalence of G x E effect or even unusual climatic factors between seasons and years [44]. In this study, the negative prediction accuracies, especially for Swift Current, could be because of trait variation between years resulting G x E as Swift Current is located at semi-arid environment, with large year-to-year variation for soil moisture and temperature. It also might be associated with lack of highly effective SNP markers for certain traits, such as dry matter yield.

Our study showed differences in the prediction accuracy of models for the same trait evaluated in two different environments, and the prediction accuracy was statistically significant for the GS models, location and their interactions for most of the traits. The prediction accuracy was moderate for forage yield related traits in Saskatoon, while it was moderate for PH and FRG in Swift Current. This difference can be attributed partly to the difference in the genotypes being used in the study and the inability of the genomic selection models used to account for G × E effect. SNP variation within-family is significantly higher than among families in crested wheatgrass [15] owing to the genetic differences among genotypes. In addition, Saskatoon (moist mixed grassland ecoregion and Dark Brown soil zone) differs in agro-climatic and soil zone from Swift Current (mixed grassland ecoregion with semi-arid condition and Brown soil zone) [72]. Similarly, in previous studies, prediction accuracy was variable between two different environments for alfalfa [35], and across seasons and years for perennial ryegrass [44]. This could be the effect of different gene actions involved in two different environments. A GS study in rice (*Oryza sativa* L.) reported reduction in prediction accuracies with incorporation of data from uncorrelated environments [73].

In this study, we also investigated the effect of SNP densities in the prediction accuracy of GS models with the use of four different SNP densities corresponding to the SNP missing levels. Our results showed increasing, decreasing and a mixture of increasing and decreasing trends of prediction accuracy with increasing SNP densities for the agro-morphological and nutritive value traits (Figs 2 and 3). Higher marker densities that are evenly distributed across the genome have been reported to improve the prediction accuracies by increasing the probability of each QTL to be in LD with at least one marker [71, 74, 75]. In contrast to this assumption, little improvement in prediction accuracy (increasing trend) with increasing SNP densities in our study could have resulted from uneven genome coverage of the GBS markers in which most of the SNPs are often generated from small portion of DNA samples, missing markers, and the imputation method implemented to reconstruct the missing markers, although a large number of SNPs are generated through the GBS [41, 76, 77]. This decreases the actual markers affecting the trait controlled by large number of QTLs, consequently limiting the prediction accuracy of the GS models. Prediction accuracy of GS in switchgrass was not affected by reducing the SNP densities to 3000 [41]. In a GS study in perennial ryegrass [78], utilizing SNP markers generated from GBS, the prediction accuracy was similar with about a threefold increase in the marker numbers. Increase in prediction accuracy for traits with complex genetic architecture, and a decrease in prediction accuracy for traits with simple genetic architecture with increasing marker density have been reported [79]. However, both decreasing and increasing trends of the prediction accuracy with increasing SNP density observed for complex traits in the present study could be explained by the genetic makeup of the traits, inability of GBS markers to cover the large genome and inability of the models to account for gene interactions. Differences in the trends of prediction accuracy for traits evaluated at Saskatoon and Swift Current could be the result of different genetic interactions in different environments. Difference in the prediction accuracy of oil content in rapeseed (*Brassica napus*) evaluated in two different years were reasoned to be influenced by pleiotropic effects of certain genetic factors in specific environments [80].

This study assessed whether SNPs generated using the GBS application in diploid and tetraploid crested wheatgrass plants could predict the GEBVs of agro-morphological and nutritive value traits in crested wheatgrass using four GS models. Overall, the moderate prediction accuracies observed for DTH, LW, PH, CD, ESV and FRG, demonstrate that a genomic selection approach could increase the genetic gain for these traits in a cost-effective way by reducing the length of the selection cycle. This approach could be utilized for the development of high-quality, high-yielding, late maturing crested wheatgrass for extended summer grazing. However, the low prediction accuracies observed for certain traits in our study might be improved by increasing the number of replications for improved phenotyping, refining the training population using a higher number of breeding lines, increasing the population size, using different genotyping approach to increase the genome coverage and using different genomic selection models that account for additive and dominant gene actions [81–83], and G x E interactions [84–86]. In addition to this, the genetic architecture of the traits under study influences the prediction accuracy of the GS models, and thus, for the traits with unknown genetic architecture in crested wheatgrass, studies are needed for the comparison of parametric models with the non-parametric models which are reported to perform better under epistatic gene effects. We foresee that the development of high-density markers with a higher level of genome coverage would enhance the use of GS applications in crested wheatgrass breeding.

## 5 Conclusions

This genomic selection analysis in combination with GBS revealed moderate predication accuracies (0.20 to 0.35) for several agro-morphological traits in crested wheatgrass breeding. This finding is encouraging for the application of GS in perennial forage crop breeding.
